# (*Z*)-4-(2,5-Di-*tert*-butyl­anilino)pent-3-en-2-one

**DOI:** 10.1107/S1600536811017296

**Published:** 2011-05-14

**Authors:** Jesús Pastrán, Andrea Ramírez, Giuseppe Agrifoglio, Anthony Linden, Romano Dorta

**Affiliations:** a Departamento de Química, Universidad Simón Bolívar, Caracas 1080A, Venezuela; b Institute of Organic Chemistry, University of Zürich, Winterthurerstrasse 190, CH-8057 Zürich, Switzerland

## Abstract

In the crystal structure of the title ketoamine, C_19_H_29_NO, the bond lengths from the N atom through the alkene group to the ketone O atom show the presence of an extensively delocalized π-system. The dihedral angle between the plane of the phenyl ring and that of the alkene component is 63.45 (7)° due to steric hindrance exerted by the *tert*-butyl groups. The mol­ecule has a *Z*-configured alkene function, which is facilitated by an intra­molecular N—H⋯O hydrogen bond between the amine and ketone groups. The mol­ecules are linked into extended chains, which run parallel to the [010] direction, by a very weak C—H⋯O inter­action between the methyl substituent of the alkene group and the ketone O atom of a neighbouring mol­ecule.

## Related literature

For the conformations of β-ketoamines, see: Pastrán *et al.* (2011 ▶); Zharkova *et al.* (2009 ▶). For reactions involving amino­ketonate complexes, see: He *et al.* (2003 ▶); Hsu, Chang *et al.* (2004 ▶); Lai *et al.* (2005 ▶); Li *et al.* (2005 ▶); Tang *et al.* (2005 ▶); Hsu, Li *et al.* (2007 ▶); Pan *et al.* (2008 ▶). For the preparation and coordination chemistry of amino­ketonate ligands, see: Jones *et al.* (1998 ▶); Shukla *et al.* (2005 ▶); Lesikar *et al.* (2008 ▶); Sedai *et al.* (2008 ▶).
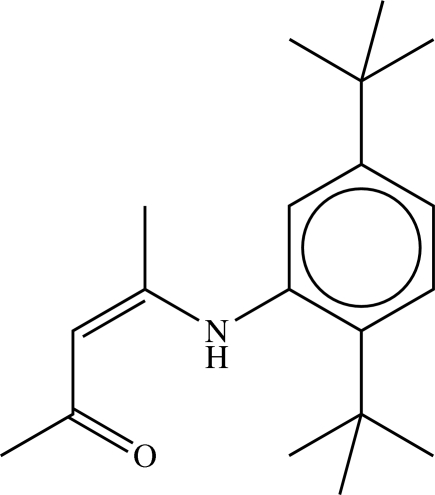


## Experimental

### 

#### Crystal data

C_19_H_29_NO
*M* *_r_* = 287.44
Monoclinic, 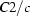

*a* = 23.7759 (5) Å
*b* = 9.0517 (2) Å
*c* = 19.3760 (4) Å
β = 120.6308 (11)°
*V* = 3588.11 (13) Å^3^
*Z* = 8
Mo *K*α radiation
μ = 0.06 mm^−1^
*T* = 160 K
0.32 × 0.25 × 0.20 mm

#### Data collection

Nonius KappaCCD area-detector diffractometer
24643 measured reflections
3153 independent reflections
2769 reflections with *I* > 2σ(*I*)
*R*_int_ = 0.035

#### Refinement

*R*[*F* ^2^ > 2σ(*F* ^2^)] = 0.044
*wR*(*F* ^2^) = 0.118
*S* = 1.04
3152 reflections
203 parameters
H atoms treated by a mixture of independent and constrained refinement
Δρ_max_ = 0.19 e Å^−3^
Δρ_min_ = −0.17 e Å^−3^

### 

Data collection: *COLLECT* (Nonius, 2000 ▶); cell refinement: *DENZO-SMN* (Otwinowski & Minor, 1997 ▶); data reduction: *DENZO-SMN* and *SCALEPACK* (Otwinowski & Minor, 1997 ▶); program(s) used to solve structure: *SHELXS97* (Sheldrick, 2008 ▶); program(s) used to refine structure: *SHELXL97* (Sheldrick, 2008 ▶); molecular graphics: *ORTEPII* (Johnson, 1976 ▶); software used to prepare material for publication: *SHELXL97* and *PLATON* (Spek, 2009 ▶).

## Supplementary Material

Crystal structure: contains datablocks I, global. DOI: 10.1107/S1600536811017296/lh5243sup1.cif

Structure factors: contains datablocks I. DOI: 10.1107/S1600536811017296/lh5243Isup2.hkl

Supplementary material file. DOI: 10.1107/S1600536811017296/lh5243Isup3.cdx

Supplementary material file. DOI: 10.1107/S1600536811017296/lh5243Isup4.cml

Additional supplementary materials:  crystallographic information; 3D view; checkCIF report

## Figures and Tables

**Table 1:** Hydrogen-bond geometry (Å, °)

*D*—H⋯*A*	*D*—H	H⋯*A*	*D*⋯*A*	*D*—H⋯*A*
N15—H15⋯O18	0.908 (17)	1.848 (17)	2.6376 (15)	144.1 (15)
C20—H201⋯O18^i^	0.98	2.52	3.474 (2)	164

Symmetry code: (i) 
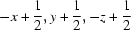
.

**Fig. 1.**
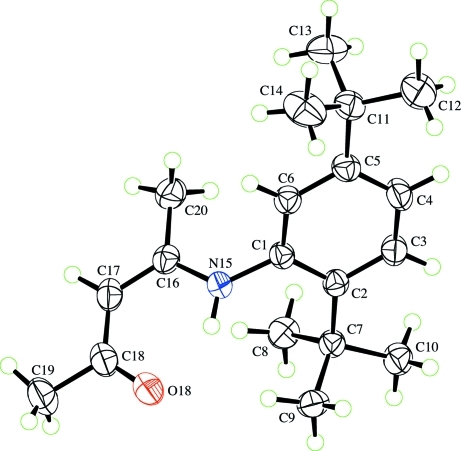
View of the molecule of (I) showing the atom-labelling scheme. Displacement ellipsoids are drawn at the 50% probability level. H atoms are represented by circles of arbitrary size.

## supplementary crystallographic information

### Comment

Anions of β-amino-α-enones are potentially useful bidentate ligands (Jones, *et al.*, 1998; Shukla, *et al.*, 2005; Hsu, Li *et al.*, 2007; Lesikar, *et al.*, 2008; Sedai, *et al.*, 2008) in stoichiometric (Hsu, Chang *et al.*, 2004) and catalytic processes (He, *et al.*, 2003; Lai, *et al.*, 2005; Li, *et al.*, 2005; Tang, *et al.*, 2005; Pan, *et al.*, 2008). Generally, β-amino-α-enones have *Z* conformations that are stabilized by intramolecular hydrogen bonds (Zharkova, *et al.*, 2009; Pastrán *et al.*, 2011). The title β-amino-α-enone was derived from 2,5-di-*tert*-butyl-aniline and acetylacetone, *via* the 4-[(-)1-phenyl-ethylamino]-pent-3-en-2-one intermediate (Lai, *et al.*, 2005). In its crystal structure, the plane of the aryl ring is twisted out of the plane spanned by the C═C double bond (defined by atoms N15, C16, C17, C18 and C20) by 63.45 (7)° due to the steric pressure exerted by the *tert*-butyl groups (Fig. 1). The bond lengths from N15 through the alkene group to the ketone O atom, O18, show the presence of an extensively delocalized π-system (Table 1). Even the C1—N15 bond is shorter than a normal single bond, despite the twist about this bond. The *Z*-configuration of the molecule facilitates the formation of an intramolecular N—H···O hydrogen bond between the amine group and the carbonyl O-atom (Table 2). The molecules are linked into extended 2_1_-symmetrical chains, which run parallel to the [010] direction, by a very weak C—H···O interaction between the methyl substituent of the alkene group and the ketone O atom of a neighbouring molecule. There are no other significant intermolecular interactions in the structure.

### Experimental

The title compound was prepared by refluxing 2,5-di-*tert*-butyl-aniline (1.14 g, 5.56 mmol) with 4-[(-)1-phenyl-ethylamino]-pent-3-en-2-one (Lai, *et al.*, 2005) (1.14 g, 5.60 mmol) in dry ethanol (30 ml) and HCl (12*M*, 0.5 ml) for 24 h. The cooled reaction mixture was treated with 1*M* K_2_CO_3_ and extracted with CH_2_Cl_2_ (3 × 10 ml). The extracts were dried over MgSO_4_, filtered, and the volatiles evaporated *in vacuo* to afford an orange oil. Methanol (2.0 ml) was added and the resulting solution was cooled to 273 K for two days to yield 0.50 g (37%) of colorless crystals (m.p. 325–327 K). ^1^H-NMR (400 MHz, CDCl_3_): δ 1.28 (s, 9H), 1.35 (s, 9H), 1.79 (s, 3H), 2.10 (s, 3H), 5.22 (s, 1H), 7.35–6.97 (m, 3H), 12.48 (s, 1H).

### Refinement

The amine H atom was located in a difference Fourier map and its position and isotropic displacement parameter were refined freely. All other H atoms were placed in geometrically idealized positions and constrained to ride on their parent atoms with C—H = 0.95 and 0.98 Å for aromatic and methyl H atoms, respectively, and with *U*_iso_(H) = 1.2*U*_eq_(C) for aromatic H atoms or 1.5*U*_eq_(C) for methyl groups. Twelve low angle reflections were excluded from the data set because they were obscured by the beam stop.

### Crystal data

**Table:**

C_19_H_29_NO	*F*(000) = 1264
*M**_r_* = 287.44	*D*_x_ = 1.064 Mg m^−^^3^
Monoclinic, *C*2/*c*	Melting point: 326 K
Hall symbol: -C 2yc	Mo *K*α radiation, λ = 0.71073 Å
*a* = 23.7759 (5) Å	Cell parameters from 3360 reflections
*b* = 9.0517 (2) Å	θ = 2.0–25.0°
*c* = 19.3760 (4) Å	µ = 0.06 mm^−^^1^
β = 120.6308 (11)°	*T* = 160 K
*V* = 3588.11 (13) Å^3^	Prism, colourless
*Z* = 8	0.32 × 0.25 × 0.20 mm

### Data collection

**Table:**

Nonius KappaCCD area-detector diffractometer	2769 reflections with *I* > 2σ(*I*)
Radiation source: Nonius FR590 sealed tube generator	*R*_int_ = 0.035
horizontally mounted graphite crystal	θ_max_ = 25.0°, θ_min_ = 3.0°
Detector resolution: 9 pixels mm^-1^	*h* = 0→28
ω scans with κ offsets	*k* = 0→10
24643 measured reflections	*l* = −23→19
3153 independent reflections	

### Refinement

**Table:**

Refinement on *F*^2^	Secondary atom site location: difference Fourier map
Least-squares matrix: full	Hydrogen site location: difference Fourier map
*R*[*F*^2^ > 2σ(*F*^2^)] = 0.044	H atoms treated by a mixture of independent and constrained refinement
*wR*(*F*^2^) = 0.118	*w* = 1/[σ^2^(*F*_o_^2^) + (0.056*P*)^2^ + 2.1259*P*] where *P* = (*F*_o_^2^ + 2*F*_c_^2^)/3
*S* = 1.04	(Δ/σ)_max_ = 0.001
3152 reflections	Δρ_max_ = 0.19 e Å^−^^3^
203 parameters	Δρ_min_ = −0.17 e Å^−^^3^
0 restraints	Extinction correction: *SHELXL97* (Sheldrick, 2008), Fc^*^=kFc[1+0.001xFc^2^λ^3^/sin(2θ)]^-1/4^
Primary atom site location: structure-invariant direct methods	Extinction coefficient: 0.0053 (10)

### Special details

**Table:**

Experimental. Solvent used: MeOH Cooling Device: Oxford Cryosystems Cryostream 700 Crystal mount: glued on a glass fibre Mosaicity (°.): 0.811 (1) Frames collected: 1331 Seconds exposure per frame: 60 Degrees rotation per frame: 0.3 Crystal-Detector distance (mm): 30.0
Geometry. All e.s.d.'s (except the e.s.d. in the dihedral angle between two l.s. planes) are estimated using the full covariance matrix. The cell e.s.d.'s are taken into account individually in the estimation of e.s.d.'s in distances, angles and torsion angles; correlations between e.s.d.'s in cell parameters are only used when they are defined by crystal symmetry. An approximate (isotropic) treatment of cell e.s.d.'s is used for estimating e.s.d.'s involving l.s. planes.
Refinement. Refinement of *F*^2^ against ALL reflections. The weighted *R*-factor *wR* and goodness of fit *S* are based on *F*^2^, conventional *R*-factors *R* are based on *F*, with *F* set to zero for negative *F*^2^. The threshold expression of *F*^2^ > σ(*F*^2^) is used only for calculating *R*-factors(gt) *etc*. and is not relevant to the choice of reflections for refinement.

### Fractional atomic coordinates and isotropic or equivalent isotropic displacement parameters (Å^2^)

**Table:**

	*x*	*y*	*z*	*U*_iso_*/*U*_eq_	
O18	0.19853 (5)	0.78576 (13)	0.11732 (6)	0.0523 (3)	
N15	0.30334 (6)	0.85624 (13)	0.25496 (6)	0.0337 (3)	
H15	0.2706 (8)	0.7964 (19)	0.2197 (10)	0.048 (4)*	
C1	0.35235 (6)	0.80923 (15)	0.33326 (7)	0.0310 (3)	
C2	0.39376 (6)	0.68963 (14)	0.34376 (7)	0.0313 (3)	
C3	0.43787 (7)	0.65278 (16)	0.42337 (8)	0.0391 (3)	
H3	0.4668	0.5722	0.4339	0.047*	
C4	0.44166 (7)	0.72794 (17)	0.48796 (8)	0.0423 (4)	
H4	0.4732	0.6982	0.5409	0.051*	
C5	0.40051 (7)	0.84563 (15)	0.47720 (8)	0.0361 (3)	
C6	0.35575 (6)	0.88316 (15)	0.39810 (8)	0.0347 (3)	
H6	0.3264	0.9625	0.3880	0.042*	
C7	0.39271 (6)	0.60546 (15)	0.27398 (7)	0.0341 (3)	
C8	0.40461 (8)	0.71202 (17)	0.22106 (9)	0.0430 (4)	
H81	0.4100	0.6554	0.1817	0.065*	
H82	0.4442	0.7699	0.2547	0.065*	
H83	0.3672	0.7788	0.1930	0.065*	
C9	0.32752 (7)	0.52365 (16)	0.22340 (9)	0.0435 (4)	
H91	0.3193	0.4603	0.2583	0.065*	
H92	0.3297	0.4628	0.1830	0.065*	
H93	0.2920	0.5957	0.1966	0.065*	
C10	0.44656 (7)	0.48728 (17)	0.30488 (9)	0.0445 (4)	
H101	0.4396	0.4153	0.3376	0.067*	
H102	0.4893	0.5344	0.3375	0.067*	
H103	0.4451	0.4368	0.2592	0.067*	
C11	0.40414 (7)	0.93389 (17)	0.54685 (8)	0.0424 (4)	
C12	0.43769 (11)	0.8466 (2)	0.62528 (9)	0.0662 (5)	
H121	0.4138	0.7544	0.6185	0.099*	
H122	0.4382	0.9056	0.6680	0.099*	
H123	0.4827	0.8237	0.6397	0.099*	
C13	0.44427 (9)	1.0737 (2)	0.55784 (11)	0.0596 (5)	
H131	0.4880	1.0459	0.5692	0.089*	
H132	0.4480	1.1316	0.6027	0.089*	
H133	0.4225	1.1330	0.5087	0.089*	
C14	0.33608 (8)	0.9797 (2)	0.52823 (10)	0.0636 (5)	
H141	0.3165	1.0458	0.4817	0.095*	
H142	0.3394	1.0310	0.5747	0.095*	
H143	0.3087	0.8916	0.5164	0.095*	
C16	0.30034 (7)	0.98782 (15)	0.22102 (8)	0.0352 (3)	
C17	0.25166 (7)	1.01571 (16)	0.14306 (8)	0.0400 (4)	
H17	0.2498	1.1113	0.1217	0.048*	
C18	0.20430 (7)	0.91006 (18)	0.09300 (8)	0.0445 (4)	
C19	0.16057 (9)	0.9464 (2)	0.00524 (9)	0.0620 (5)	
H191	0.1762	0.8945	−0.0262	0.093*	
H192	0.1614	1.0532	−0.0026	0.093*	
H193	0.1158	0.9152	−0.0125	0.093*	
C20	0.35276 (8)	1.09906 (17)	0.26778 (9)	0.0468 (4)	
H201	0.3460	1.1426	0.3093	0.070*	
H202	0.3511	1.1769	0.2316	0.070*	
H203	0.3955	1.0505	0.2931	0.070*	

### Atomic displacement parameters (Å^2^)

**Table:**

	*U*^11^	*U*^22^	*U*^33^	*U*^12^	*U*^13^	*U*^23^
O18	0.0518 (7)	0.0519 (7)	0.0380 (6)	−0.0019 (5)	0.0118 (5)	0.0034 (5)
N15	0.0348 (6)	0.0330 (6)	0.0282 (6)	0.0030 (5)	0.0123 (5)	0.0014 (5)
C1	0.0304 (7)	0.0321 (7)	0.0287 (6)	−0.0007 (5)	0.0138 (5)	0.0030 (5)
C2	0.0322 (7)	0.0309 (7)	0.0314 (7)	−0.0005 (5)	0.0168 (6)	0.0022 (5)
C3	0.0427 (8)	0.0388 (8)	0.0343 (7)	0.0104 (6)	0.0185 (6)	0.0048 (6)
C4	0.0438 (8)	0.0483 (9)	0.0290 (7)	0.0087 (7)	0.0143 (6)	0.0062 (6)
C5	0.0404 (7)	0.0380 (8)	0.0312 (7)	−0.0021 (6)	0.0192 (6)	0.0000 (6)
C6	0.0357 (7)	0.0347 (7)	0.0345 (7)	0.0027 (6)	0.0184 (6)	0.0008 (6)
C7	0.0368 (7)	0.0338 (7)	0.0314 (7)	0.0033 (6)	0.0172 (6)	0.0009 (5)
C8	0.0534 (9)	0.0438 (8)	0.0408 (8)	0.0013 (7)	0.0304 (7)	0.0013 (6)
C9	0.0437 (8)	0.0367 (8)	0.0464 (8)	−0.0017 (6)	0.0202 (7)	−0.0079 (6)
C10	0.0462 (8)	0.0463 (9)	0.0406 (8)	0.0115 (7)	0.0218 (7)	0.0004 (6)
C11	0.0484 (8)	0.0472 (9)	0.0322 (7)	0.0013 (7)	0.0211 (7)	−0.0024 (6)
C12	0.0964 (14)	0.0672 (12)	0.0355 (9)	0.0107 (11)	0.0340 (9)	0.0006 (8)
C13	0.0714 (11)	0.0555 (11)	0.0541 (10)	−0.0118 (9)	0.0336 (9)	−0.0184 (8)
C14	0.0579 (10)	0.0914 (14)	0.0497 (9)	0.0034 (10)	0.0334 (8)	−0.0173 (9)
C16	0.0418 (7)	0.0337 (7)	0.0352 (7)	0.0071 (6)	0.0233 (6)	0.0017 (6)
C17	0.0472 (8)	0.0397 (8)	0.0350 (7)	0.0123 (6)	0.0223 (6)	0.0079 (6)
C18	0.0437 (8)	0.0530 (10)	0.0339 (8)	0.0131 (7)	0.0177 (7)	0.0047 (7)
C19	0.0629 (11)	0.0701 (12)	0.0359 (8)	0.0130 (9)	0.0126 (8)	0.0070 (8)
C20	0.0573 (9)	0.0379 (8)	0.0450 (8)	−0.0018 (7)	0.0258 (7)	0.0025 (6)

### Geometric parameters (Å, °)

**Table:**

O18—C18	1.2541 (19)	C10—H102	0.9800
N15—C16	1.3447 (18)	C10—H103	0.9800
N15—C1	1.4280 (16)	C11—C14	1.525 (2)
N15—H15	0.908 (17)	C11—C12	1.528 (2)
C1—C6	1.3888 (18)	C11—C13	1.533 (2)
C1—C2	1.4066 (18)	C12—H121	0.9800
C2—C3	1.3935 (18)	C12—H122	0.9800
C2—C7	1.5411 (17)	C12—H123	0.9800
C3—C4	1.387 (2)	C13—H131	0.9800
C3—H3	0.9500	C13—H132	0.9800
C4—C5	1.388 (2)	C13—H133	0.9800
C4—H4	0.9500	C14—H141	0.9800
C5—C6	1.3911 (18)	C14—H142	0.9800
C5—C11	1.5324 (19)	C14—H143	0.9800
C6—H6	0.9500	C16—C17	1.3797 (19)
C7—C8	1.5354 (19)	C16—C20	1.496 (2)
C7—C10	1.5365 (18)	C17—C18	1.418 (2)
C7—C9	1.5374 (19)	C17—H17	0.9500
C8—H81	0.9800	C18—C19	1.510 (2)
C8—H82	0.9800	C19—H191	0.9800
C8—H83	0.9800	C19—H192	0.9800
C9—H91	0.9800	C19—H193	0.9800
C9—H92	0.9800	C20—H201	0.9800
C9—H93	0.9800	C20—H202	0.9800
C10—H101	0.9800	C20—H203	0.9800
			
C16—N15—C1	126.49 (12)	C14—C11—C12	109.23 (14)
C16—N15—H15	110.4 (10)	C14—C11—C5	110.88 (12)
C1—N15—H15	123.0 (10)	C12—C11—C5	111.88 (13)
C6—C1—C2	121.74 (12)	C14—C11—C13	108.59 (15)
C6—C1—N15	117.22 (12)	C12—C11—C13	108.44 (14)
C2—C1—N15	121.01 (11)	C5—C11—C13	107.73 (12)
C3—C2—C1	114.89 (12)	C11—C12—H121	109.5
C3—C2—C7	121.29 (12)	C11—C12—H122	109.5
C1—C2—C7	123.81 (11)	H121—C12—H122	109.5
C4—C3—C2	123.21 (13)	C11—C12—H123	109.5
C4—C3—H3	118.4	H121—C12—H123	109.5
C2—C3—H3	118.4	H122—C12—H123	109.5
C3—C4—C5	121.62 (13)	C11—C13—H131	109.5
C3—C4—H4	119.2	C11—C13—H132	109.5
C5—C4—H4	119.2	H131—C13—H132	109.5
C4—C5—C6	115.96 (12)	C11—C13—H133	109.5
C4—C5—C11	123.27 (12)	H131—C13—H133	109.5
C6—C5—C11	120.75 (12)	H132—C13—H133	109.5
C1—C6—C5	122.57 (13)	C11—C14—H141	109.5
C1—C6—H6	118.7	C11—C14—H142	109.5
C5—C6—H6	118.7	H141—C14—H142	109.5
C8—C7—C10	107.28 (11)	C11—C14—H143	109.5
C8—C7—C9	110.23 (11)	H141—C14—H143	109.5
C10—C7—C9	106.40 (11)	H142—C14—H143	109.5
C8—C7—C2	110.44 (11)	N15—C16—C17	120.22 (13)
C10—C7—C2	111.39 (11)	N15—C16—C20	118.68 (12)
C9—C7—C2	110.96 (11)	C17—C16—C20	121.02 (13)
C7—C8—H81	109.5	C16—C17—C18	123.81 (14)
C7—C8—H82	109.5	C16—C17—H17	118.1
H81—C8—H82	109.5	C18—C17—H17	118.1
C7—C8—H83	109.5	O18—C18—C17	123.28 (13)
H81—C8—H83	109.5	O18—C18—C19	118.33 (15)
H82—C8—H83	109.5	C17—C18—C19	118.33 (15)
C7—C9—H91	109.5	C18—C19—H191	109.5
C7—C9—H92	109.5	C18—C19—H192	109.5
H91—C9—H92	109.5	H191—C19—H192	109.5
C7—C9—H93	109.5	C18—C19—H193	109.5
H91—C9—H93	109.5	H191—C19—H193	109.5
H92—C9—H93	109.5	H192—C19—H193	109.5
C7—C10—H101	109.5	C16—C20—H201	109.5
C7—C10—H102	109.5	C16—C20—H202	109.5
H101—C10—H102	109.5	H201—C20—H202	109.5
C7—C10—H103	109.5	C16—C20—H203	109.5
H101—C10—H103	109.5	H201—C20—H203	109.5
H102—C10—H103	109.5	H202—C20—H203	109.5
			
C16—N15—C1—C6	65.87 (17)	C3—C2—C7—C10	−2.30 (18)
C16—N15—C1—C2	−116.26 (15)	C1—C2—C7—C10	176.39 (12)
C6—C1—C2—C3	−0.26 (19)	C3—C2—C7—C9	116.04 (14)
N15—C1—C2—C3	−178.03 (12)	C1—C2—C7—C9	−65.27 (16)
C6—C1—C2—C7	−179.02 (12)	C4—C5—C11—C14	−143.77 (16)
N15—C1—C2—C7	3.21 (19)	C6—C5—C11—C14	37.95 (19)
C1—C2—C3—C4	−0.5 (2)	C4—C5—C11—C12	−21.6 (2)
C7—C2—C3—C4	178.27 (13)	C6—C5—C11—C12	160.16 (14)
C2—C3—C4—C5	0.8 (2)	C4—C5—C11—C13	97.53 (17)
C3—C4—C5—C6	−0.2 (2)	C6—C5—C11—C13	−80.74 (17)
C3—C4—C5—C11	−178.53 (14)	C1—N15—C16—C17	176.87 (12)
C2—C1—C6—C5	0.8 (2)	C1—N15—C16—C20	0.26 (19)
N15—C1—C6—C5	178.71 (12)	N15—C16—C17—C18	−2.8 (2)
C4—C5—C6—C1	−0.6 (2)	C20—C16—C17—C18	173.76 (13)
C11—C5—C6—C1	177.79 (13)	C16—C17—C18—O18	6.9 (2)
C3—C2—C7—C8	−121.41 (14)	C16—C17—C18—C19	−170.40 (14)
C1—C2—C7—C8	57.28 (16)		

### Hydrogen-bond geometry (Å, °)

**Table:**

*D*—H···*A*	*D*—H	H···*A*	*D*···*A*	*D*—H···*A*
N15—H15···O18	0.908 (17)	1.848 (17)	2.6376 (15)	144.1 (15)
C20—H201···O18^i^	0.98	2.52	3.474 (2)	164

Symmetry codes: (i) −*x*+1/2, *y*+1/2, −*z*+1/2.
